# Zn‐doped MnO_x_ nanowires displaying plentiful crystalline defects and tunable small cross-sections for an optimized volcano-type performance towards supercapacitors

**DOI:** 10.1186/s11671-023-03933-2

**Published:** 2023-12-04

**Authors:** Geyse A. C. Ribeiro, Scarllett L. S. de Lima, Karolinne E. R. Santos, Jhonatam P. Mendonça, Pedro Macena, Emanuel C. Pessanha, Thallis C. Cordeiro, Jules Gardener, Guilhermo Solórzano, Jéssica E. S. Fonsaca, Sergio H. Domingues, Clenilton C. dos Santos, André H. B. Dourado, Auro A. Tanaka, Anderson G. M. da Silva, Marco A. S. Garcia

**Affiliations:** 1 Departamento de Química, Centro de Ciências Exatas E Tecnologia, Universidade Federal Do Maranhão (UFMA), São Luís, MA Brazil; 2 Departamento de Engenharia Química E de Materiais-DEQM, Pontifícia Universidade Católica Do Rio de Janeiro (PUC-Rio), Rio de Janeiro, RJ Brazil; 3 Centro de Ciências Exatas E Tecnologia, Universidade Estadual Do Norte Fluminense Darcy Ribeiro (UENF), Rio de Janeiro, RJ Brazil; 4 Center for Nanoscale Systems, School of Engineering and Applied Sciences, Harvard University, Cambridge, USA; 5 Mackenzie Institute for Advanced Research in Graphene and Nanotechnologies – MackGraphe, Mackenzie Presbyterian University, São Paulo, SP Brazil; 6 Departament of Physics, Universidade Federal Do Maranhão (UFMA), São Luís, MA Brazil; 7 São Carlos Institute of Chemistry, Universidade de São Paulo (USP), São Carlos, SP Brazil

**Keywords:** Nanowires, MnO_2_, Zn, Supercapacitors, Oxygen vacancies, Surface defects

## Abstract

MnO_x_-based nanomaterials are promising large-scale electrochemical energy storage devices due to their high specific capacity, low toxicity, and low cost. However, their slow diffusion kinetics is still challenging, restricting practical applications. Here, a one-pot and straightforward method was reported to produce Zn-doped MnO_x_ nanowires with abundant defects and tunable small cross-sections, exhibiting an outstanding specific capacitance. More specifically, based on a facile hydrothermal strategy, zinc sites could be uniformly dispersed in the α-MnO_x_ nanowires structure as a function of composition (0.3, 2.1, 4.3, and 7.6 wt.% Zn). Such a process avoided the formation of different crystalline phases during the synthesis. The reproducible method afforded uniform nanowires, in which the size of cross-sections decreased with the increase of Zn composition. Surprisingly, we found a volcano-type relationship between the storage performance and the Zn loading. In this case, we demonstrated that the highest performance material could be achieved by incorporating 2.1 wt.% Zn, exhibiting a remarkable specific capacitance of 1082.2 F.g^−1^ at a charge/discharge current density of 1.0 A g^−1^ in a 2.0 mol L^−1^ KOH electrolyte. The optimized material also afforded improved results for hybrid supercapacitors. Thus, the results presented herein shed new insights into preparing defective and controlled nanomaterials by a simple one-step method for energy storage applications.

**Supplementary Information:**

The online version contains supplementary material available at 10.1186/s11671-023-03933-2.

## Introduction

Struggles to balance the imminent energy crisis and global climate warming are part of long and challenging attempts to develop alternative energy supplies and in-depth research into energy storage systems [1, 2]. At this point, with the knowledge regarding actions for substantial changes, it is undeniable that renewable energy source improvements are directly related to storing energy issues. Supercapacitors are in the spotlight in this scenario due to their high power yields and increased storage capacity compared to conventional capacitors [3]. Scientists worldwide focus on the following materials for supercapacitor electrodes: carbonaceous metals, oxides/hydroxides, and conducting polymers [4–6]. Such materials are very different in nature, offering diverse characteristics: carbon-based materials deliver high power with low energy density, while the other two give lower kinetics than the former materials, but their storage energy in pseudo-Faradaic processes, which can be similar to batteries, which is a vital feature for storage devices [7].

Recently, pseudocapacitance materials like metal sulfides, carbides, and oxides have attracted a substantial audience in energy storage applications [8]. Their large specific capacitances and high energy densities match most current electronic devices' high power and energy density requirements. In this case, metal oxides (NiO, MnO_2_, Co_3_O_4_, RuO_2_, etc.) have been demonstrated to be the most promising candidates for pseudocapacitance electrodes [9]. Among these candidates, MnO_2_ is particularly interesting due to its high availability, low cost, low toxicity, high theoretical capacitance, and excellent capacitive performance in water-based electrolytes, making them promising for supercapacitor electrodes [10]. In this case, the charge storage mechanism in MnO_2_ is typically based on fast faradaic redox reactions that either occur at the electrode surface or inside the bulk of the materials over an appropriate range of potentials. Therefore, to promote high capacitive performance, a large surface area and fast ion/electron transport by the electrode material are crucial in these systems.

Although MnO_2_ has been one of the most investigated materials due to its favorable electrochemical activities, its specific capacitance values are still far below the theoretical value. The poor electronic conductivity and limited surface area are responsible for low specific capacitance values in bulk MnO_2_-based electrodes. In this regard, researchers have been continuously trying to improve the performances of MnO_2_ by a variety of means, e.g., by synthesizing high surface area nanomaterials displaying controlled sizes and shapes (nanowires, nanoflowers, nanorods, etc.) [11–13], combining with conductive components (carbon black, carbon nanotubes, graphene, etc.), doping different metals (Al, Fe, Zn, Co, Ni, and Ag), and defect engineering to enhance the electron transport properties to attain a reasonable capacitance value [10].

Among these strategies to improve the electrochemical performances of MnO_2_, several authors have directed their efforts to manipulate the physicochemical features of nanomaterials by controlling their size, shape, morphology, and surface area. The manipulation of such parameters causes various phenomena that can be explained due to the control over the nanoparticle size, shape, composition, and structure, which strongly affect their properties, making nanoparticle control an efficient strategy for maximizing performance [14]. In particular, one-dimensional (1D) MnO_2_ nanowires have attracted significant attention in catalysis and energy storage for the following reasons: (i) higher specific surface areas relative to those of commercial samples; (ii) crystal growth along highly catalytically active crystallographic directions, leading to high performances; and (iii) easily accessible surface by gas and liquid substrates due to their porous structures [15]. Furthermore, decreasing the cross-section diameters in nanowires can be presented as a successful approach for further enhancing promising properties of MnO_2_ nanowires (surface area, oxygen vacancies, ratio of Mn^3+^/Mn^4+^ ions at the surface, etc.).

In this context, nanoengineering crystalline defects (such as oxygen and metal vacancies) is an efficient strategy for designing nanomaterials with new tunable features. Generally, it can be done by doping MnO_2_ with a second metal (Ni, Co, Zn, Cu, Ag, Fe, etc.), increasing the availability of active and adsorption sites and the number of oxygen vacancies at the nanomaterials' surface that consequently enable favorable ion diffusion and electron charge-transfer. These features have been extensively used for catalysis, sensors, and electronic devices [16]. However, electrochemical issues have the advantage of having attainable oxidation states.

Research is actively underway to enhance the capacity and promote commercial applications of cathode materials in rechargeable potassium-ion batteries [17]. Nevertheless, there is a growing consensus that more substantial efforts are required in the realm of supercapacitors, particularly in the context of ion intercalation in non-noble metal-based candidates. Intriguingly, cation substitution has been explored to evaluate the pseudocapacitive performance of supercapacitors, yielding impressive results for asymmetric supercapacitors (achieving an energy density of 29.3 W h kg^−1^ even at a remarkably high-power density of 8000 W kg^−1^)[18]. Additionally, the creation of oxygen-deficient hydrated potassium manganese oxide for high-energy, flexible magnesium-ion supercapacitors has been made possible through a sodium preintercalation-induced process [19].

Furthermore, the introduction of sulfur-induced oxygen vacancies has been investigated to expedite the redox reactivity within CuCo_2_S_4_ hollow nanoarchitectures, resulting in flexible solid-state asymmetric supercapacitors that offer a substantial energy density of 61.4 W h kg^−1^ at 750 W kg^−1^. Consequently, there remains a significant need for extensive research in energy storage, with a clear emphasis on the critical role of vacancy generation. In this case, the transition metals selection to prepare the binary oxides must be well-thought-out to guarantee good performance with stability.

Zinc oxide (ZnO) has emerged as an appropriate material for energy storage issues for similar reasons described for MnO_2_; also, Zn composites seem to maximize the utilization of electrochemical sites [20]. However, one must remember that an essential feature in electrocatalysis is the charge transfer at the electrode interface and the electrolyte [21]. Thus, apart from the composition, it is still lacking in the literature more fundamental investigations on the structure-properties relationship, such as the effect of physical modifications of MnO_2_-based nanomaterials on the performance improvement of supercapacitors.

Therefore, we prepared uniform α-MnO_x_ nanowires doped with Zn by incorporating different amounts of zinc nitrate precursor during the formation of MnO_x_ nanowires under hydrothermal conditions, exhibiting an outstanding specific capacitance. Remarkably, zinc sites showed uniform dispersion on the α-MnO_x_ nanowire structure, depending on the composition (0.3, 2.1, 4.3, and 7.6 wt.% Zn), resulting in an elevation of oxygen vacancies concentration and a reduction in the cross-sectional sizes as the Zn composition increased. Such findings directly reflect the materials' storage performance, showing a volcano-type relationship.

## Experimental section

### Materials and instrumentation

All the chemicals utilized in this study were of analytical quality. Manganese sulfate monohydrate (MnSO_4_·H_2_O, 99%, Sigma–Aldrich), potassium permanganate (KMnO_4_, 99%, Sigma–Aldrich), zinc nitrate (Zn(NO_3_)_2_·6H_2_O, 98%, Sigma–Aldrich), ethanol (95%, Vetec), potassium hydroxide (KOH, 99%, Isofar), Poly(vinylidene fluoride) (PVDF, average molecular weight ~ 534,000, Sigma–Aldrich), Super P carbon black (Super P, > 99%, Alfa Aesar), and 1-Methyl-2-pyrrolidinone (NMP, 99.5%, Sigma–Aldrich) were employed without any additional purification steps. Scanning electron microscopy (SEM) images were obtained using a JEOL field emission gun microscope JSM 7800F (JEOL, Tokyo, Japan) at 10 kV with a lower electron detector (LED). To prepare the samples, 0.01 g of the material was dispersed in 10 mL of ultrapure water (resistivity of 18.2 M Ω cm^−1^, Millipore®, Billerica, USA) and sonicated for 10 min to suspend the material in the solvent. Subsequently, the aqueous suspension containing the nanostructures was deposited onto a silicon wafer and dried under ambient conditions.

Transmission electron microscope (TEM) images were acquired using a HITACHI HT 7800 (HITACHI, Tokyo, Japan) operated at 120 kV and a Tecnai FEI G20 (Thermo Fisher Scientific, Massachusetts, EUA) operated at 200 kV. A drop-casting method was also employed to prepare the nanomaterials for TEM analysis. The nanostructure suspension was deposited onto a carbon-coated copper grid and allowed to dry under ambient conditions. The Zn content dispersed on MnO_x_ nanowires was measured using inductively coupled plasma optical emission spectrometry (ICP-OES) with a Spectro Arcos instrument (SPECTRO Analytical Instruments GmbH, Kleve, Germany). The crystalline structure of the MnO_x_ and Zn-doped MnO_x_ nanowires was examined by X-ray diffraction (XRD) using a Bruker D8 Discover diffractometer (Bruker Corporation, Massachusetts, EUA) in a 2θ range of 10°–90° with a step size of 0.02◦ using Cu Kα radiation. The samples were deposited on a sapphire background-free support and allowed to dry before XRD analysis.

The textural properties of Zn-doped MnO_x_ nanowires were investigated through N_2_-adsorption isotherms recorded at − 196 °C using a NovaTouch 2-LX instrument (Anton Paar GmbH, Graz, Austria). The nanomaterials (100 mg) were degassed for 3 h at 130 °C before analysis. The specific surface areas were determined using the Brunauer–Emmett–Teller (BET) equation from an adsorption isotherm generated in a relative pressure range of 0.07 < P/P_o_ < 0.3. The total pore volume was calculated from the amount of N_2_ adsorbed at a relative pressure close to unity, and the average pore diameter was determined using the Barrett-Joyner-Halenda (BJH) method from the N_2_ desorption isotherms.

Electron paramagnetic resonance (EPR) spectra were collected using a Bruker Elexsys E500 spectrometer (Bruker Corporation, Massachusetts, EUA) operating at Xband (~ 9.8 GHz) with a DC magnetic field ranging from 200 to 500 mT, an AC magnetic field with a frequency of 100 kHz, and a modulation amplitude of 0.5 mT. The DC magnetic field was calibrated with a Bruker standard strong pitch sample. In this procedure, 2 mg of the material was placed into a quartz tube inserted into the high-sensitivity (cylindrical) resonant cavity with a microwave power of 15.89 mW.

X-ray photoemission spectroscopy (XPS) spectra were acquired with a Scienta Omicron ESCA + spectrometer system equipped with an EA 125 hemispherical analyzer and an XM 1000 monochromated X-ray source (Scientia Omicron, Uppsala, Sweden) using Al Kα (1486.7 eV) as the X-ray source. Data analysis was performed using CasaXPS processing software version 2.3.15 (Casa Software Ltd., Teignmouth, UK).

### α-MnO_x_ and Zn-doped-MnO_x_ nanowires preparation

The α-MnO_x_ nanowires were synthesized via a hydrothermal method [22]. In a typical procedure, 0.4 g of MnSO_4_·H_2_O and 1.0 g of KMnO_4_ were dissolved in 30 mL of deionized water. This solution was transferred into a 100 mL Teflon-lined stainless-steel autoclave and maintained at 140 °C for 19 h. Subsequently, the system was gradually cooled to room temperature, and the resulting dark product was subjected to three rounds of washing with 15 mL of ethanol and three rounds of washing with 15 mL of water, accomplished through sequential centrifugation and removal of the supernatant. Following this, the material was dried at 80 °C for 6 h in an open-air environment. The same protocol was employed to synthesize Zn-doped MnO_x_ nanowires displaying different Zn compositions. Thus, the exact amounts of the Mn precursors were used, as described before; however, various Zn(NO_3_)_2_^.^ 6H_2_O quantities (17, 87, 170, and 348 mg) were mixed into the system separately, using the same volume of deionized water (30 mL). After that, the washing and drying processes were followed as defined above.

### Electrochemical measurements

The electrochemical measurements were performed using an Autolab PGSTAT 302N potentiostat (Metrohm, Herisau, Switzerland) with NOVA 2.0 processing software. The working electrode comprised nickel foam (1.2 mm thick, 1 cm × 1 cm) modified with MnO_x_ or the Zn-doped MnO_x_ nanowires samples and nickel foil (0.125 mm thick, 1 mm × 3 mm). Its preparation followed the procedure described here: the nickel foam was etched in 3.0 mol L^−1^ HCl solution, washed thoroughly with isopropanol/water, and dried at 60 °C for 12 h; then, some pieces of the foam were cut (1 cm^2^), weighted, and saved for later utilization. In the next step, a slurry of each sample was prepared: the sample, the conducting Super P, and the PVDF binder dissolved in NMP solvent were mixed at a weight ratio of 8:1:1. At that moment, the previously etched pieces of foam were used to place 20 µL of the obtained slurry; after, the set was dried overnight, and, after that, we determined the mass of the active material by weighting the foam, now modified with the samples. By pressing it with a nickel wire as a current collector, the electrode was ready to be used [23]. The electrode was inserted into a single-compartment glass cell with Pt gauze as the auxiliary electrode and Ag|AgCl (KCl saturated) as the reference electrode, against which all potentials shall be referenced here. The data were acquired in 2.0 mol L^−1^ potassium hydroxide solution media. The Cyclic Voltammetry (CV) essays were recorded with the N_2_-saturated electrolyte, with different scan rates (5 to 80 mV s^−1^), in a potential range of 0.0 V to 0.5 V. The Galvanostatic Charge–Discharge (GCD) tests were conducted under different current densities (1, 2, 5, 10, and 20 A g^−1^).

The specific capacity (C_s_, F g^−1^) of the studies herein performed was calculated according to Eq. (1) [24]:

1 $${\text{C}}_{{\text{s}}} = \frac{{{\text{I}}.\Delta {\text{t}}}}{{{\text{m}}.\Delta {\text{v}}}}$$

where I (A) corresponds to the current, ∆t (s) is the discharging time, m (g) denotes the mass of material used for the working electrode preparation, and ∆V (V) is the potential window.

The Coulombic efficiency (η) was obtained using Eq. (2) [24]:

2 $$\eta = \frac{{t_{d} }}{{t_{c} }} \times 100{\text{\% }}$$

where t_d_ and t_c_ are the discharging and charging times, respectively.

The hybrid supercapacitor tests were performed in a two-electrode system. In typical analyses, the nickel foam was loaded with the Zn-doped MnO_x_ nanowires samples and served as a positive electrode; activated carbon was used as a negative one. The charge stored (q) by each electrode depended on the following Eq. (3) [24]:

3 $$q=m\bullet {C}_{s}\bullet \Delta v$$

in which C_s_ is specific capacitance, ∆V is the potential window of charge–discharge behavior, and m is the mass of the active electrode material. The active material weight loaded on the cathode for the hybrid supercapacitor to obtain Q +  = Q − was obtained using the Eq. (4) [25]:

4 $$\frac{{m}_{+}}{{m}_{-} }=\frac{{Q}_{+}}{{Q}_{-}}$$

where m_+_ and m_-_ denote the weights of the materials loaded on cathode and anode electrodes, respectively. Equations (5) and (6) were used to obtain the energy density (ED) and the power density (PD) [26]:

5 $$ED=\frac{1}{2}\bullet {C}_{s}{\left(\Delta V\right)}^{2}$$

6 $$PD = 3600 \times \frac{ED}{{\Delta t}}$$

where C_s_ is the specific capacitance (F g^−1^) obtained in the GCD tests for the hybrid supercapacitor, ∆V (V) is the potential range between the cathode and anode, and ∆t (s) is the time of discharge of the device. All the tests were carried out at room temperature.

## Results and discussion

**Fig. 1:**
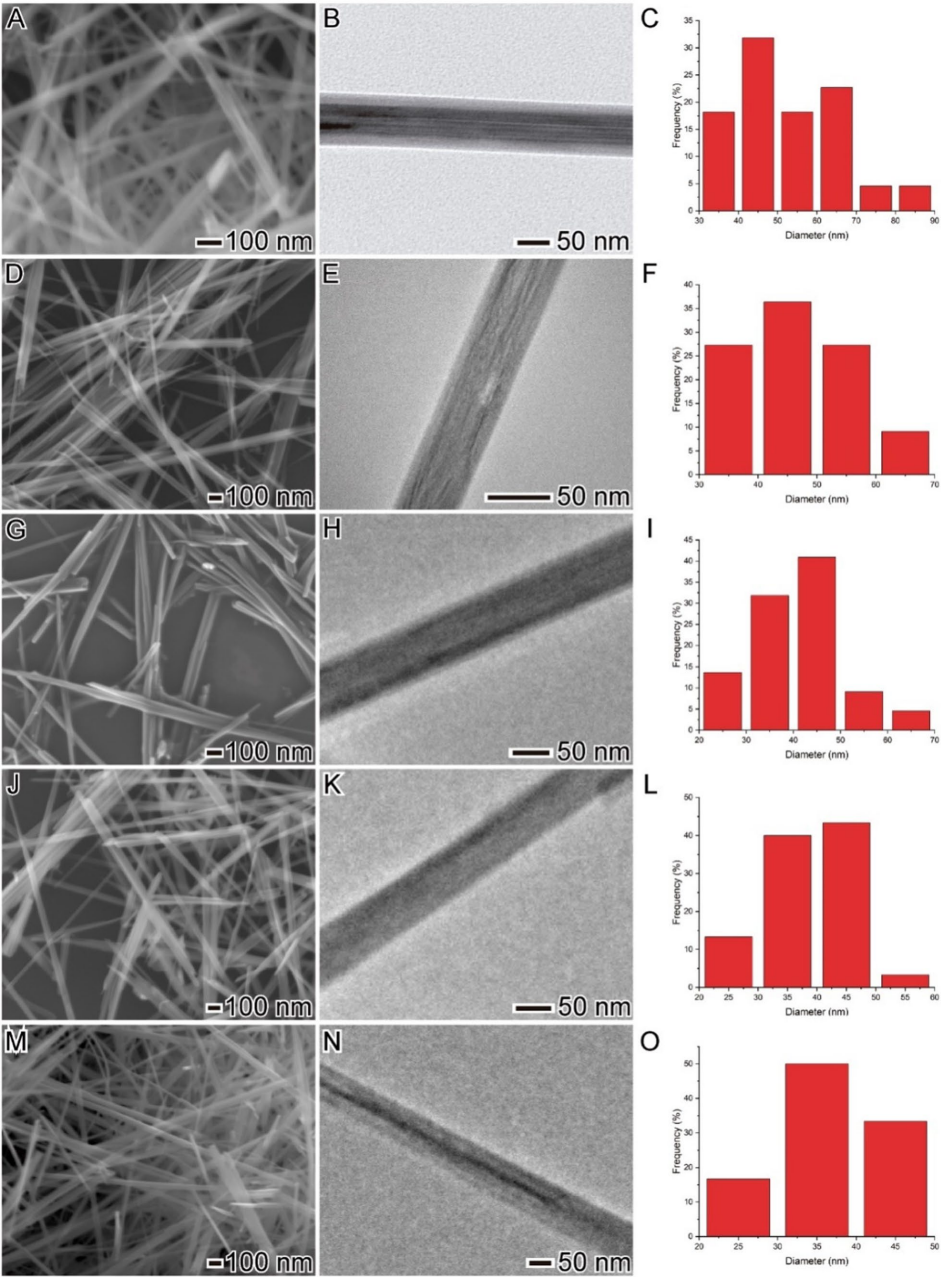
SEM (A, C, E, G, and I), TEM (B, D, F, H, and J), and histograms (C, F, I, L, and O) images of the materials obtained by a hydrothermal method. (A-C) MnO_x_, (D-F) 0.3%, (G-I) 2.1%, (J-L) 4.3%, and (M–O) 7.6 wt.% Zn-doped MnO_x_ nanowires, respectively

Our investigations started with the hydrothermal synthesis of MnO_x_ nanowires using MnSO_4_ (reducing agent) and KMnO_4_ (oxidizing agent) as metal precursors and water as solvent at 110 °C for 24 h. Such a synthetic procedure was chosen once this method leads to defects on the surface of the nanomaterial that could directly impact its electrochemical behavior. Also, this procedure is very simple and can provide "clean" nanostructures without capping ligands or related compounds. The nanowires displayed well-defined shapes and uniform sizes, 51 ± 14 nm in width and > 1 µm in length, as shown in Fig. 1A–C**.** In the next step, we modified the classical hydrothermal synthesis of MnO_x_ nanowires by adding different amounts of Zn(NO_3_)_2._6H_2_O (described in the experimental section) to the synthesis procedure. We used Zn amounts to obtain 1.0, 5.0, 10.0, and 20.0 wt.% Zn. However, using the ICP-OES technique, the following content was obtained by increasing the Zn precursor: 0.3, 2.1, 4.3, and 7.6 wt.% Zn.

**Fig. 2:**
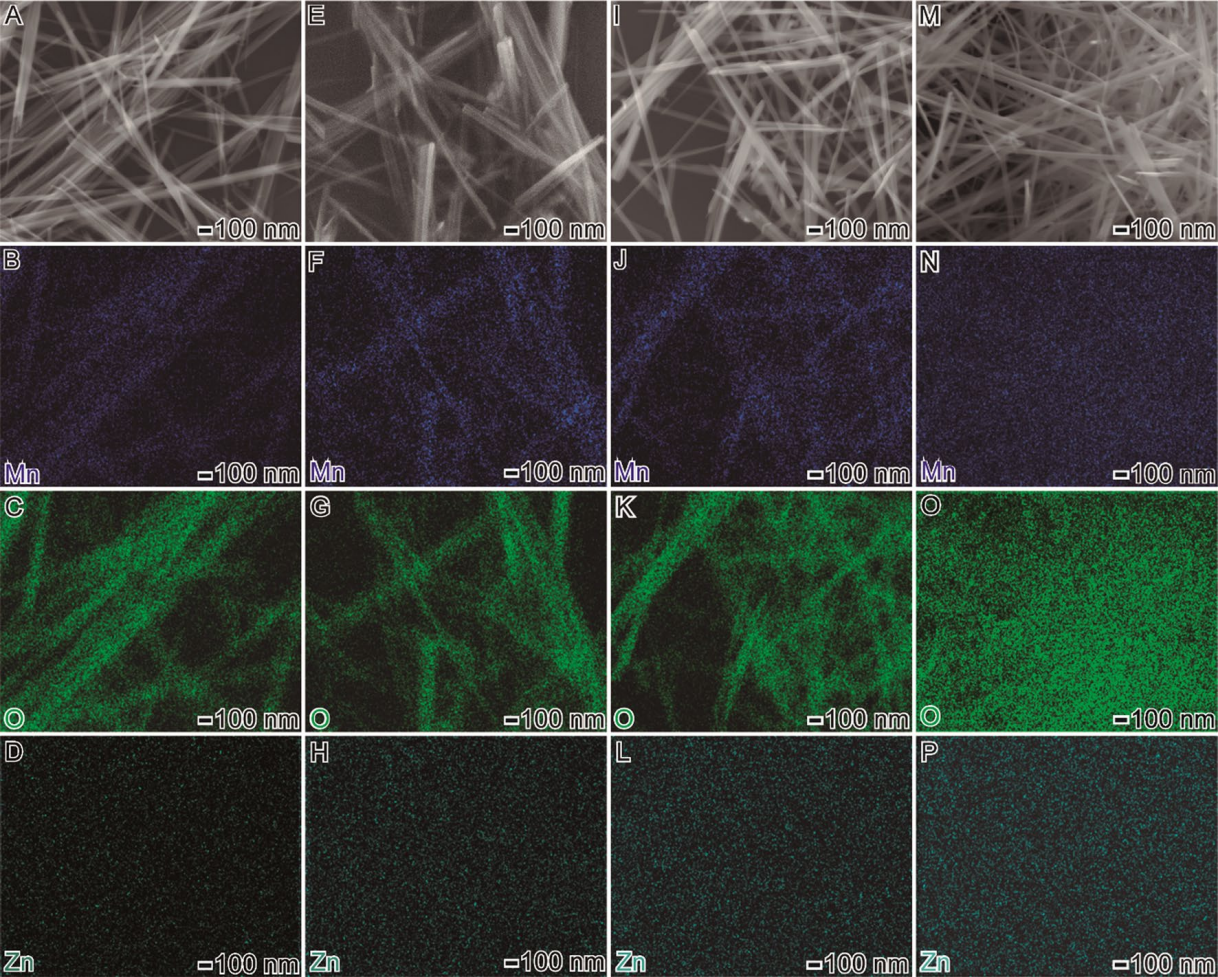
SEM images of the 0.3 (A), 2.1 (E), 4.3 (I), and 7.6 (M) wt.% Zn-doped MnO_x_ nanowires. The elemental mapping colors are: blue (Mn), green (O), and drank green (Zn). Specifically, (B-D), (F–H), (J-L), and (N-P) are the mapping of the 0.3, 2.1, 4.3, and 7.6 wt% of Zn doped-MnO_x_ nanowires, respectively

Interestingly, in all the cases, Zn-doped MnO_x_ nanowires presented well-defined shapes and uniform sizes; however, their cross-section diameters depended on the zinc composition. More specifically, the Zn-doped nanowires displayed cross-section diameters of 47 ± 10 nm, 41 ± 11 nm, 39 ± 8 nm, 37 ± 6 nm in width, and > 1 µm in length, as shown in Fig. 1D–F, G−I, J–L, and M–O, for 0.3, 2.1, 4.3, and 7.6 wt.% Zn-doped MnO_x_ nanowires, respectively. Additional file 1: Fig. S1 presents HRTEM images of individual 0.3, 2.1, 4.3, and 7.6 wt.% of Zn doped MnO_x_ nanowires, revealing that they are structurally uniform, with mixed growth directions along the [110], [100], and [310] axis. We also performed STEM-EDX spectrum imaging for all the Zn-doped MnO_x_ nanowires to investigate the Mn, O, and Zn elemental distribution, as shown in Fig. 2. Interestingly, it confirmed the uniform distribution of Mn and O. Also, it is clear the gradual increase in intensity in the signal of Zn at the SEM–EDS elemental mapping images of 0.3, 2.1, 4.3, and 7.6 wt.% Zn-doped MnO_x_ nanowires, in agreement with ICP-OES results.

After synthesizing the Zn-doped MnO_x_ nanowires, we examined their textural and structural properties, which could influence their storage performances. Thus, the textural properties obtained by N_2_-physisorption analysis are presented in Table S1. The specific surface area of samples increased with the increase in zinc composition, displaying 105, 107, 132, 144, and 135 m^2^ g^−1^ for 0.3, 2.1, 4.3, and 7.6 wt.% Zn-doped MnO_x_ samples, respectively, in agreement with the TEM analyses that showed a decrease in the cross-section diameters of nanowires as the content of zinc increased. The Zn-doped MnO_x_ nanowires also displayed a smaller average BJH pore diameter as the zinc content increased. The representative adsorption isotherms (we have chosen the MnO_x_ and 2.1 wt.% Zn-doped MnO_x_ nanowires samples) – Figure S2 – corresponded to type IV (IUPAC classification), typical of mesoporous materials. The shape of the hysteresis loop corresponded to type H3, with well-defined loops that did not level off at relative pressures close to the saturation vapor pressure. Such characteristics are important for the preparation of supercapacitor electrodes once they can provide enhanced ion accessibility, increased surface area, stability, and durability.

The XRD results of the 0.3, 2.1, 4.3, and 7.6 wt.% Zn-doped MnO_x_ nanowires are shown in Figure S3. The samples exhibit similar XRD patterns, indexed mainly as α-MnO_2_ tetragonal crystal phase with I4/m space group (JCPDS Card No: 44–0141) [27]. Specifically, 2θ values at 12.8, 18.0, 28.7, 37.5, 41.9, 49.8, 56.0, 60.1, 65.3°, and 69.5°, ascribed to (110), (200), (310), (211), (301), (411), (600), (521), (002), and (541) crystal planes, were clearly observed for the MnO_x_ nanowires sample. Interestingly, a significant broadening and slight shift of all diffraction peaks could be observed when the composition of Zn increased. Such results suggest that zinc sites could be lightly and uniformly dispersed onto MnO_x_ nanowires’ structure, avoiding the formation of different crystalline phases (only α-MnO_2_ was detected). Thus, such a procedure avoided the structural transformation of α-MnO_2_ nanowires during the synthesis. Also, the obtained data suggest that Zn is doped into the oxide lattice, leading to some distortion (evidenced by the observed shifts) and poor structure crystallinity. Poor crystallinity is preferred for supercapacitor applications because the structure can provide an easy way for most active ions to penetrate.

**Fig. 3:**
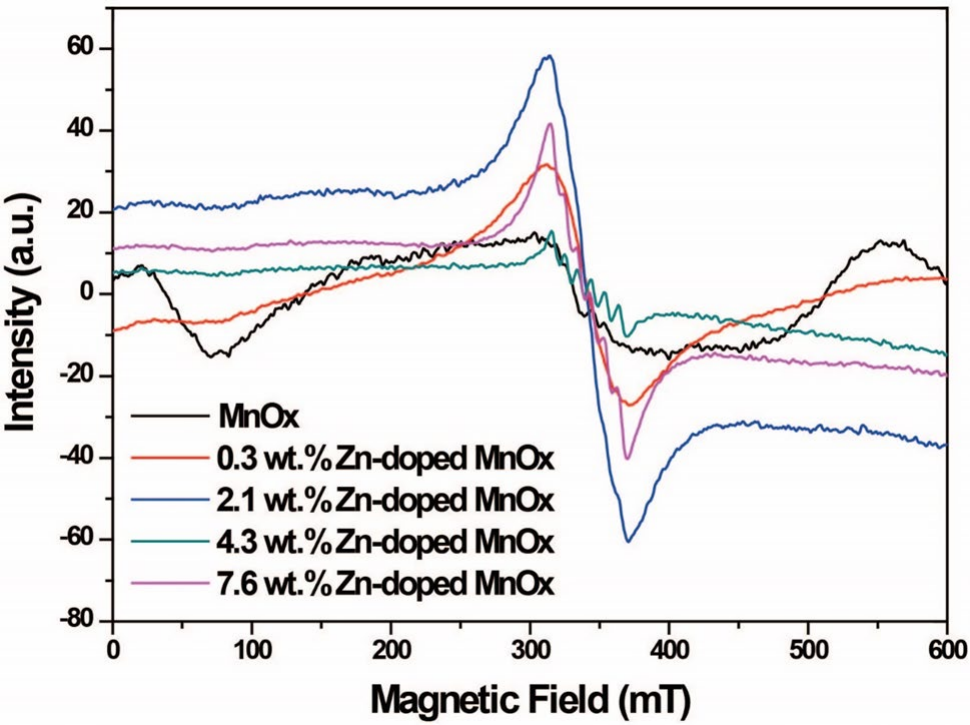
EPR spectra for the MnO_x_ and Zn-doped MnO_x_ nanowires

The EPR spectra of the MnO_x_ and Zn-doped MnO_x_ nanowires were investigated to understand the influence of Zn doping on oxygen vacancy generation and electronic structure modification. Figure 3 shows the EPR spectra of the samples. The intensity of the EPR signal and linewidth increases with Zn addition from 0.3 to 2.1 wt.% Zn. Such signals are attributed to Mn^4+^. As Kakazey et al. demonstrated, the spectrum's broadening from 0.3 to 2.1 wt.% Zn is due to exchange interactions between Mn^4+^ ions and can also be ascribed to the partial substitution of Mn^4+^ for Mn^3+^ ions [28]. Therefore, based on EPR spectra, the higher Mn^3+^/Mn^4+^ ratio was achieved for the sample 2.1 wt.% Zn-doped MnO_x_ nanowires. In addition, as previously demonstrated by Dang et al*.*, the higher the Mn^3+^/Mn^4+^ ratio in MnO_2_-based structures, the better performance towards tailored supercapacitors due to increased MnO_2_ conductivity [29]. They demonstrated that this improved performance could be ascribed to the so-called double exchange interaction mechanism (Mn^3+^-O-Mn^4+^). In this mechanism, there is an interaction between Mn and O orbits in which electrons from Mn^3+^ could migrate to Mn^4+^ using O 2p orbits as a bridge. It was also demonstrated that Mn^4+^ to Mn^3+^ substitution in the MnO_2_ lattice induces the formation of oxygen vacancies [28, 29].

Interestingly, as Zn content addition increases, the EPR signal and linewidth decrease, and so does the sextet pattern; the characteristic hyperfine splitting of Mn^2+^ becomes more evident, as shown in Figure S4 [30–32]. Thus, based on our findings, we hypothesize that both the Mn^3+^/Mn^4+^ ratio and oxygen vacancies concentration are increasing, which could play a pivotal and synergistic role in Zn-doped MnO_x_ nanowires' improved performance up to 2.1 wt.% Zn. Hereafter, as Zn content increases, an adverse effect on the supercapacitor's performance associated with Mn^2+^ presence becomes significant.

**Fig. 4:**
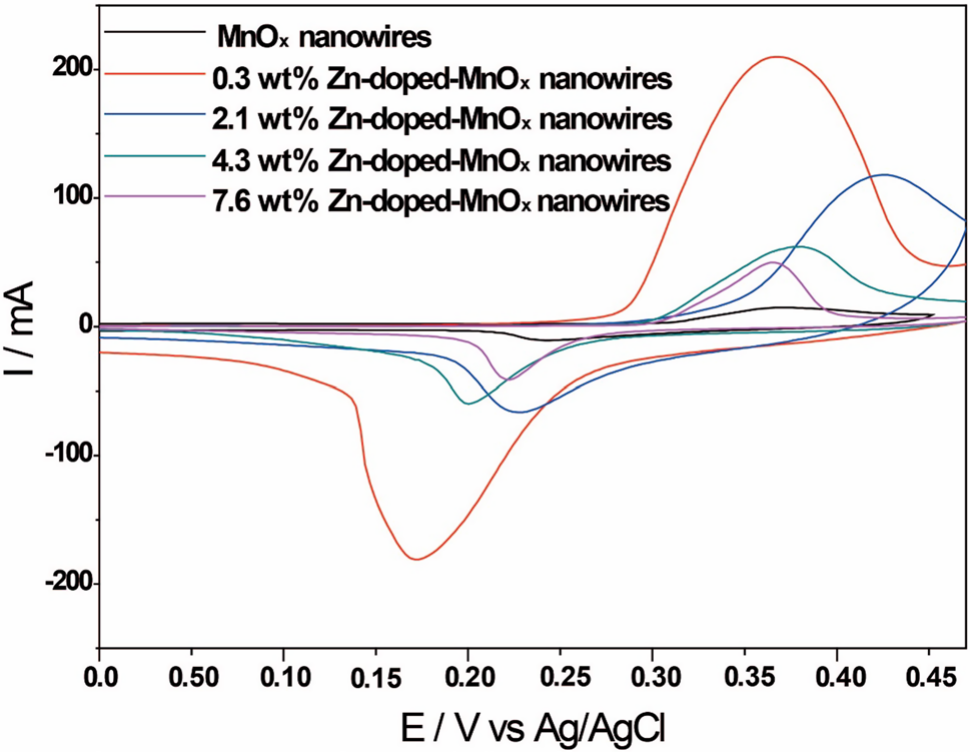
CV curves of MnO_2_ nanowires and 0.3, 2.1, 4.3, and 7.6 wt.% Zn-doped MnO_x_ nanowires at a 40 mVs^−1^ scan rate (KOH 2.0 M)

In the next step, we focused our attention on applying the MnO_x_ nanowires and 0.3, 2.1, 4.3, and 7.6 wt.% Zn-doped MnO_x_ counterparts as electrodes for storage applications. CV curves were performed to analyze the materials' electrochemical performance, as shown in Fig. 4. The electrochemical characteristics of the nanowires (doped and undoped) are indicated by the anodic and cathodic peaks in the CV curves in the range of − 0.2–0.47 V in 2.0 mol L^−1^ KOH.^26^ There are two faradaic peaks for all the electrodes, one related to an oxidation process, observed during the positive going scan, centered around 0.35 V, and another, a reduction one, observed during the negative going scan, centered around 0.20 V. The potential peak is not the same for all materials and is related to the redox couple Mn_2_O_3_/MnOOH. The fact that the peaks are separated by at least 140 mV (7.6% material) shows that the reaction is not happening just on the material surface; there is at least a mass transport step in which some species approach or leave the electric double layer. In addition, the process cannot be called reversible due to this large peak separation, which is larger than the ∽60/n mV separation window. However, for the 0.3 and 4.3 wt.%, the ratio between the current peaks is close to 1, indicating that the process can be quasi-reversible. The lower concentration of Zn ions in the MnO_x_ nanowires structure hampers its clear identification in the curves for the Zn-doped MnO_x_ materials, which reflects the XRD results that considered a single manganese oxide phase. However, once ICP-OES confirmed the existence of Zn species, it makes us believe that the following reaction occurred:

$$\left( {{\text{Zn}},{\text{ Mn}}} \right)_{{3}} {\text{O}}_{{4}} + {\text{ H}}_{{2}} {\text{O }} + {\text{ OH}}^{ - } \rightleftharpoons {\text{ZnOOH }} + {\text{ MnOOH }} + {\text{ e}}^{ - }$$

$${\text{ZnCOOH }} + {\text{ MnOOH }} + {\text{2OH}}^{ - } \rightleftharpoons {\text{ZnO}}_{{2}} + {\text{ MnO}}_{{2}} + {\text{ 2 H}}_{{2}} {\text{O }} + {\text{ 2e}}^{ - }$$

One can notice that the CV currents for the doped materials are much higher than the observed for pure MnO_x_ nanowires, indicating that the doping process enhanced the electrical conductivity of the materials. Interestingly, however, this current gain does not follow an expected trend, i.e., the increment in the Zn content did not provide a current increase. Instead, the 0.3 wt.% Zn-doped MnO_x_ material showed higher values and potential shifts (more negative potential for oxidation and reduction events than the other electrodes), followed by the 2.1 wt.% Zn-doped MnO_x_ counterpart, with a shift to a more positive potential for oxidation and reduction processes. The other two materials presented lower currents, suggesting that the doping affected their conductivity. It is important to note that the peak position correlates with the population of oxygen vacancies. For instance, the material containing 0.3 wt.% Zn exhibited the lowest vacancy population, resulting in the most negative peak potential. Conversely, the material with 2.1 wt.% Zn, possessing the highest vacancy concentration, displayed the most positive potential peak. Consequently, the other two materials, with lower vacancy levels approaching those of the 0.3 wt.% Zn material, exhibited intermediate potential peaks during the positive potential scan. Given that oxygen vacancies indicate an excess of electrons or, in other words, act as n-type dopants, it is reasonable to expect that the most heavily "doped" material (in this case, with more vacancies) would have the most positive oxidation potential, creating a significant barrier for oxidative behavior. Additionally, the same material showed the most positive reduction potential, indicative of the lowest reductive barrier, as anticipated for an n-type semiconductor.

We also performed the CV of the materials to evaluate the effect of different scan rates (Figure S5) for the dopped materials. We could observe reduction peaks shifting to more negative potentials with the scan rate increase, while the oxidation peaks shift to more positive ones for all the Zn-doped MnO_x_ nanowires, indicating a quasi-reversible feature of the redox couples. Figure S6 shows the peak current density versus the square root of the scan rate for all the materials, which suggests that diffusion-controlled reactions occur during the electrochemical process. For most materials, the slopes are similar, meaning that the diffusion of the same species limits the reaction. In the case of 0.3% material, the slope is 10 times higher, meaning the diffusion is facilitated in this electrode. The diffusion-limited species is probably the K^+^_(aq)_, responsible for the charge compensation process. The small porous diameter observed for these materials, around 2.5 nm, is in the same order of magnitude as the solvated K^+^ diameter, around 0.7 nm.

**Fig. 5:**
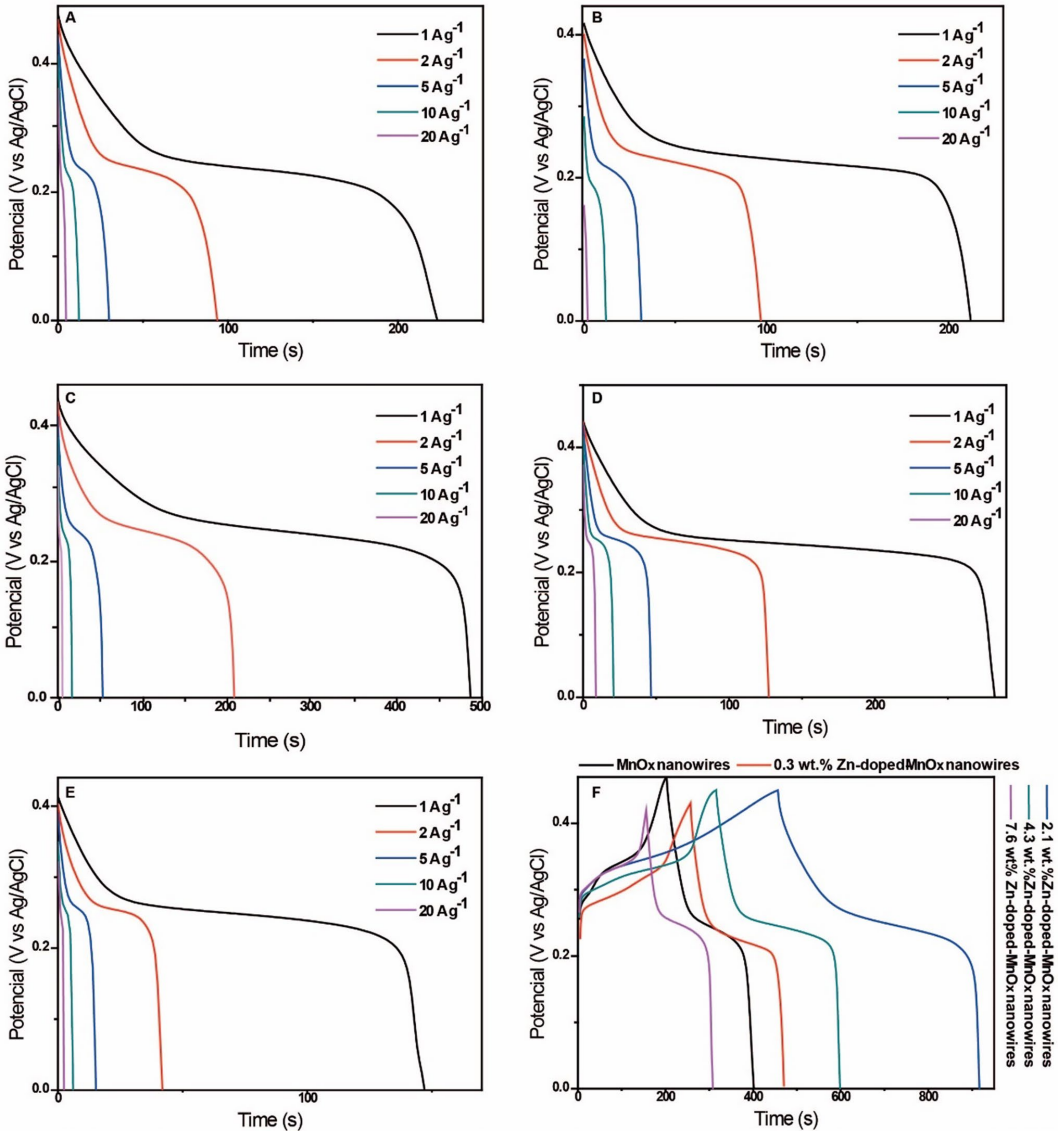
GCD curves of **A** MnO_x_, **B** 0.3 wt.% Zn, **C** 2.1 wt.% Zn, **D** 4.3 wt.% Zn, and **E** 7.6 wt.% Zn nanowires at different current densities. **F** Comparison of all the electrodes at 1 A g.^−1^

Figure 5 shows the GCD curves of the doped and undoped nanowires-based electrodes at a potential range of 0.0 to 0.45 V in 2.0 mol L^−1^ KOH electrolyte at different discharge current densities of 1.0, 2.0, 5.0, 10.0, and 20.0 A g^−1^. All the samples have only one plateau, consistent with the CV curves in Fig. 4. This plateau is not expected for electrochemical capacitors since the charge and discharge processes are not triangular, being more related to a battery-type process. For the electrode observed between battery and supercapacitor behavior, the terms "supercapattery", or intercalation pseudocapacitance are common in the literature. The profiles in the GCD measurements align with the shape expected for binary metal oxide supercapatteries. This behavior is normally related to the electrolyte and material structure and is considered a consequence of the nanostructuration of the electrode, which can make a material expected to be a supercapacitor in the bulk form behave as a battery in the nanostructured form and vice-versa. We obtained interesting specific capacities (SCs), which decreased with the increase of current density. Specifically, we had: MnO_x_ nanowires with 432.6 F g^−1^ (1 A g^−1^), 379.6 F g^−1^ (2 A g^−1^), 316.3 F g^−1^ (5 A g^−1^), 265.3 F g^−1^ (10 A g^−1^), and 204.1 F g^−1^ (20 A g^−1^); 0.3 wt.% Zn-doped-MnO_x_ nanowires with 497.7 F g^−1^ (1 A g^−1^), 451.2 F g^−1^ (2 A g^−1^), 372.1 F g^−1^ (5 A g^−1^), 302.3 F g^−1^ (10 A g^−1^), and 139.5 F g^−1^ (20 A g^−1^); 2.1 wt.% Zn-doped-MnO_x_ nanowires with 1082.2 F g^−1^ (1 A g^−1^), 928.8 F g^−1^ (2 A g^−1^), 600.0 F g^−1^ (5 A g^−1^), 340.9 F g^−1^ (10 A g^−1^), and 266.7 F g^−1^ (20 A g^−1^); 4.3 wt.% Zn-doped-MnO_x_ nanowires with 608.9 F g^−1^ (1 A g^−1^), 560.0 F g^−1^ (2 A g^−1^), 511.1 F g^−1^ (5 A g^−1^), 466.7 F g^−1^ (10 A g^−1^), 400.0 F g^−1^ (20 A g^−1^), and; 7.6 wt.% Zn-doped-MnO_x_ nanowires with 345.2 F g^−1^ (1 A g^−1^), 204.8 F g^−1^ (2 A g^−1^), 178.6 F g^−1^ (5 A g^−1^), 166.7 F g^−1^ (10 A g^−1^), and 142.9 F g^−1^ (20 A g^−1^). The Coulombic efficiencies achieved were higher than 93% for the samples in all the current densities. Figure 5F compares the study of the charge storage capacity of the synthesized electrode materials at 1 A g^−1^. The curves are virtually symmetrical, representing reversible pseudo-faradic reactions between the electrode and electrolyte.

Interestingly, a volcano-type relationship between the storage performance and the Zn loading was observed (Figure S7). Then, it is clear that we achieved an optimum Zn content for the improvement of the storage properties of the material. Above its quantity, there is a decrease in the capacity of the material. It is important to highlight that we decided not to perform characterizations after using the electrodes once previous literature showed that similar materials, after electrochemical cycles, undergo carbon deposition and Mn, O-containing nanoflakes over it, which hinder a conclusive observation of the modification of the as-prepared nanowires [13].

At this point, we could observe that supercapacitors for MnO_x_ nanowires in the literature at 1 A g^−1^ are lower than the one presented herein for our Zn-doped MnO_x_ nanowires. For example, for pure MnO_x_ nanowires, Yin et al. obtained 180 F g^−1^, while Mahmood et al. acquired 337.5 F g^−1^ [33, 34]. Undoubtedly, the material's shape and mesopore structure can improve electron transport and ion diffusion [35, 36]; however, the Zn addition seems to be the most important feature of the material's performance once the surface area is similar to the MnO_x_ nanowires. Thus, the Zn species appear to aid electron transport due to synergetic issues, as observed when Co ions are used to synthesize MnO_x_ nanowires [37, 38].

**Fig. 6:**
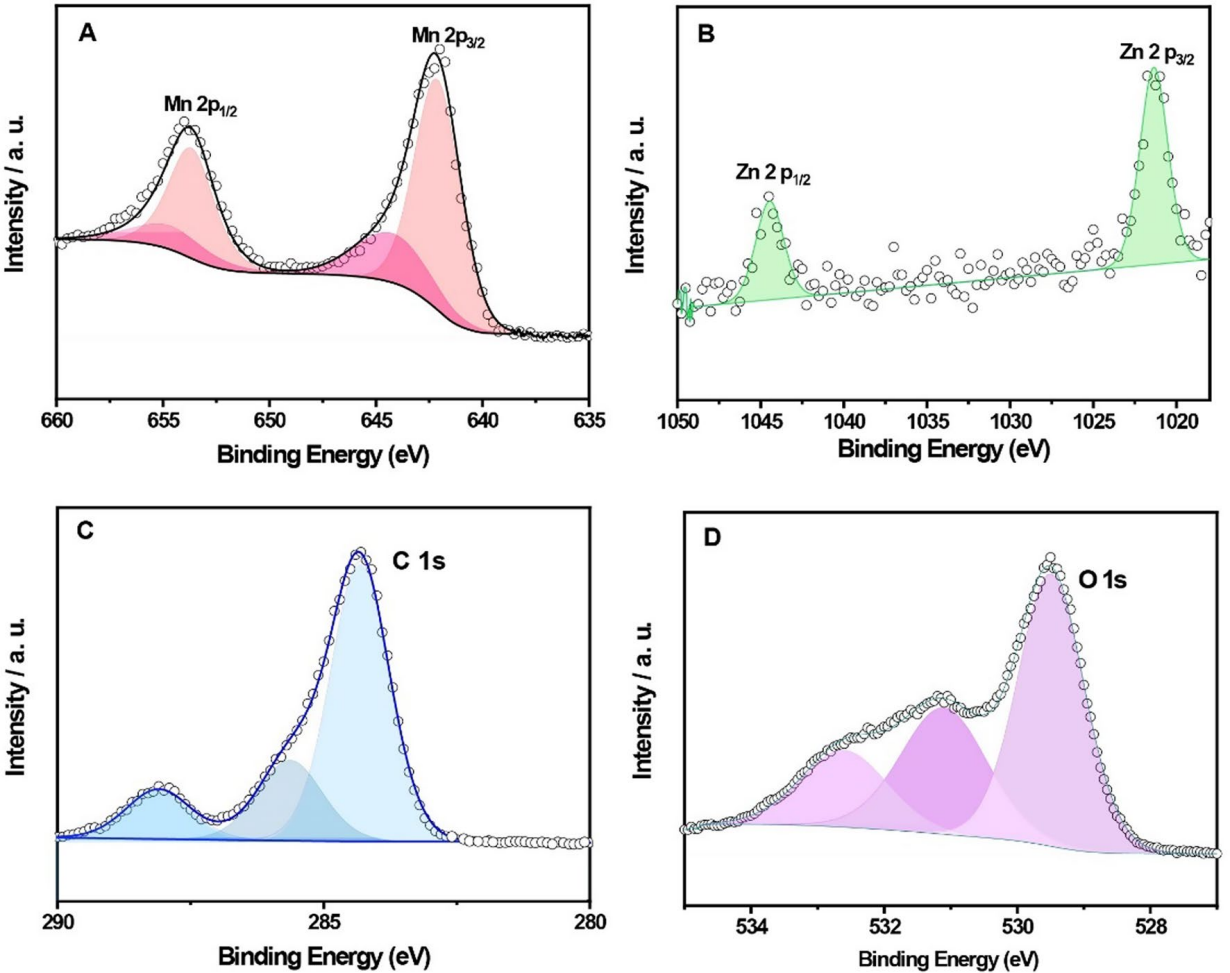
High-resolution XPS spectra of **A** Mn, **B** Zn, **C** C, and **D** O for the 2.1 wt.% Zn-doped MnO_x_ nanowires sample

The elements' chemical states and bonding natures in MnO_x_ and 2.1 wt.% Zn-doped-MnO_x_ nanowires were also investigated by XPS to shed some light on their differences. Figure S8 shows the low-resolution spectrum of the doped material, showing only the expected elements: Mn, O, Zn, and C (and a small signal regarding K from the metal precursor). Figure S9A shows the MnO_x_ nanowires' high-resolution XPS spectrum, which shows peaks centered at 653.8 and 642.2 eV, related to Mn 2p_3/2_ and Mn 2p_1/2_, respectively. An asymmetric Gaussian/Lorentzian fitting deconvolved them into four sub-peaks; two are assigned to the B.E. of Mn^3+^ (642.1 and 653.6 eV) and two to Mn^4+^ (643.5 e 654.9 eV). As shown in Fig. 6A, two fitted peaks centered at 653.9 and 642.1 eV, ascribed to Mn 2p_3/2_ and Mn 2p_1/2_, respectively, are observed for the 2.1 wt.% Zn-doped-MnO_x_, very similar to the observed for the unmodified MnO_x_ nanowires. Also, the four sub-peaks ascribed to the B.E. of Mn^3+^ (642.1 and 653.7 eV) and two to Mn^4+^ (644.2 e 654.6 eV) were detected. However, The ratio of Mn^3+^/Mn^4+^ was increased from 1.6 (MnO_x_ nanowires) to 2.9 (2.1 wt.% Zn-doped-MnO_x_ nanowires), indicating that oxygen vacancy concentration increases after in the sample with Zn doping, which follows the EPR, that shows an increasing content of Mn^3+^ in the material with 2.1 wt.% Zn. For the 2.1 wt.% Zn-doped MnO_x_ sample, we evaluated the Zn 2p region of the XPS, which showed two peaks centered at B.E. of 1021.4 and 1044 eV, ascribed for Zn 2p_3/2_ and Zn 2p_1/2_ of Zn^2+^, respectively (Fig. 6B) [39]. For the same sample, we display the C 1s spectrum (Fig. 6C); the 284.3, 285.6, and 288.1 eV peaks are associated with C–C, C–O, and O–C = O, respectively.

However, it is essential to analyze the O1s spectra for the MnO_x_ nanowires (Figure S9B) and 2.1 wt.% Zn-doped MnO_x_ nanowires (Fig. 6D). The MnO_x_ nanowires presented only two deconvoluted peaks at 529.6 and 531.4 eV. It can be seen that the three sub-peaks at 532.4 eV, 531.2 eV, and 530.1 eV belong to chemically adsorbed water (Oads), oxygen vacancy (Ov), and lattice oxygen (OL), respectively, for the doped sample. Thus, remarkable differences in the oxygen XPS show how the dopping process changed the surficial material characteristics.

However, XPS is not the most accurate technique to evaluate oxygen vacancies, so the EPR was performed herein. Thus, based on the results of EPR and XPS analyses, the formation of O_v_ vacancies could be explained as follows: during the doping and formation of manganese oxide, the Mn^4+^ species present in MnO_x_ can be replaced by Zn^2+^ sites, leading to charge disparity in the oxide structure. This phenomenon leads to a weakening of the Mn–O bond. Therefore, punctual defects such as oxygen vacancies are expected to form in the crystal lattice to compensate for this charge inequity and maintain the nanomaterial's electrical stability. These conclusions directly relate to the XPS data presented here: the Zn^2+^ can contribute to the material's electrochemical performance.

**Fig. 7:**
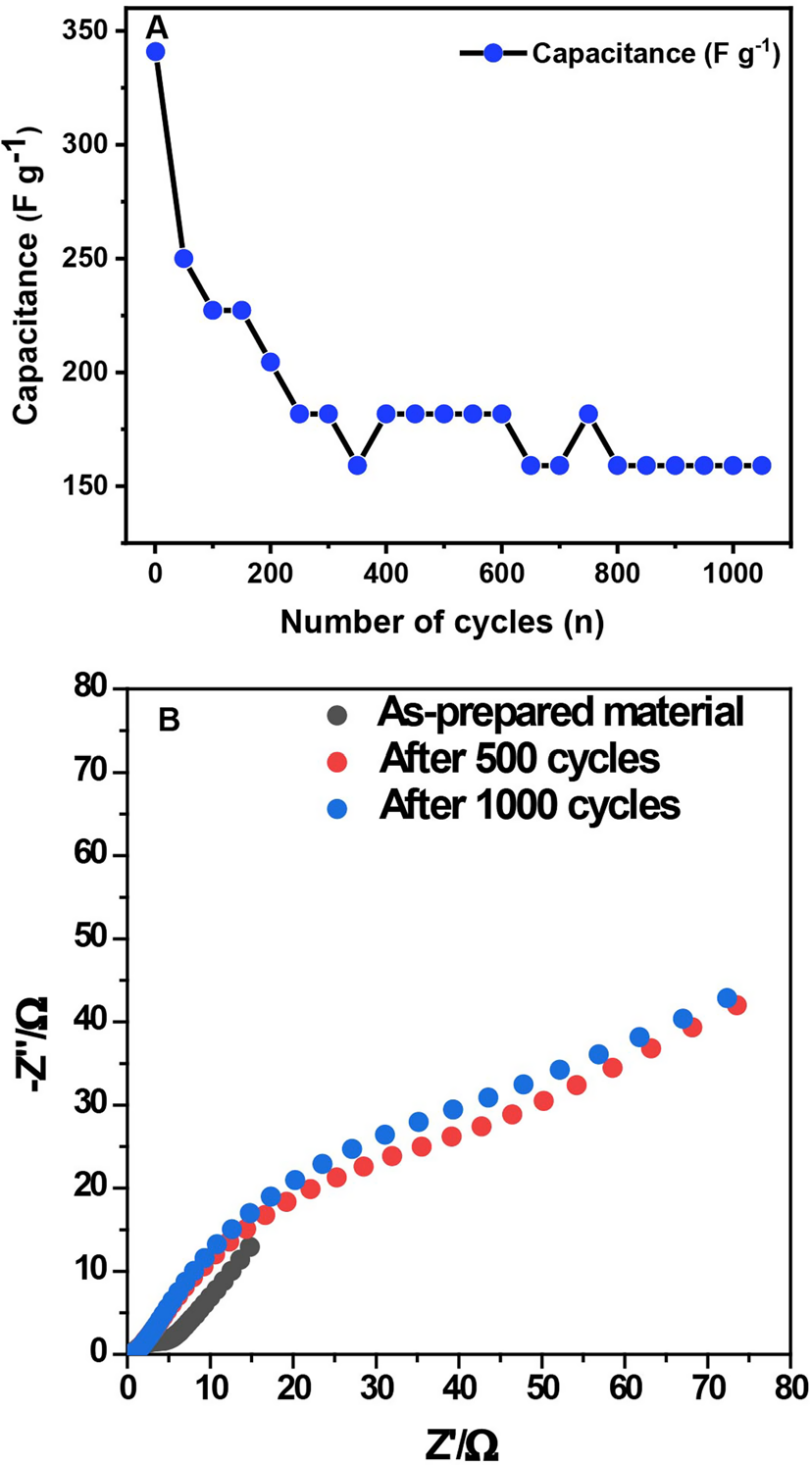
**A** Specific capacitances vs. number of cycles at 10 A g^−1^. **B** EIS analysis

By choosing the optimized doping, we obtained further Information on the 2.1 wt.% Zn-doped-MnO_2_ nanowires' cycling stability by performing 1000 charge–discharge cycles at 10 A g^−1^. According to Fig. 7A, we can notice that the capacitance retention decreases, although its Coulomb efficiency remained at 83.3%. The material experienced a considerable drop in its specific capacitance from the first to the two-hundredth cycle. This cycle achieved stabilization, staying with a capacitance of 160.5 F g^−1^ (the first test performed 340.9 F g^−1^). Thus, the material remained at 47% of its initial capacitance. Some relevant considerations are necessary regarding these results: during the cycles, irreversible redox reactions can occur, causing damage, inactivation, or loss of active sites; mechanical stress from electrolyte ions intercalation/deintercalation to counterbalance the overall charge can happen, lowering the efficiency of the material; also, insufficient active species utilization can occur due to high current densities reached in the experiments [40].

To evaluate these issues further, we performed EIS analyses at open circuit potential in 2.0 mol L^−1^ KOH electrolyte before (as-prepared), during (after 500 cycles), and after the 1000 cycles (Fig. 7B). None of the plots in Fig. 7B shows a perfect line parallel to the imaginary axis. This indicates that the system cannot be interpreted as just a resistor in series with a capacitor, so at least a constant phase element (CPE) would be needed. The fact that for the three cases, some inclination to an "infinite" semi-circle is present would suggest that at least one-time constant (a CPE/capacitor in parallel with a resistor) in series with a resistor (electrolyte resistor, R_e_) would be needed to interpret this system. For the "as-prepared" electrode, it can be seen that the data tend to have higher imaginary parts and smaller real ones, as expected for a high capacitive contribution. After some cycling, a linear region can be seen, and the slope for this is equivalent to 0.5, meaning that a mass transport limitation due to diffusion is gaining importance. The capacitance/resistive contribution is changing, as seen in Fig. 7A; however, the time constant itself seems to be kept close to the same value, so the decrease in capacitance is probably followed by an increment in resistance. After 1000 cycles, small changes are observed compared with the 500 cycles measurement, as the time-constant related points are observed to be around the same values and the diffusion-related data presented, again, 0.5 as slope.

**Fig. 8:**
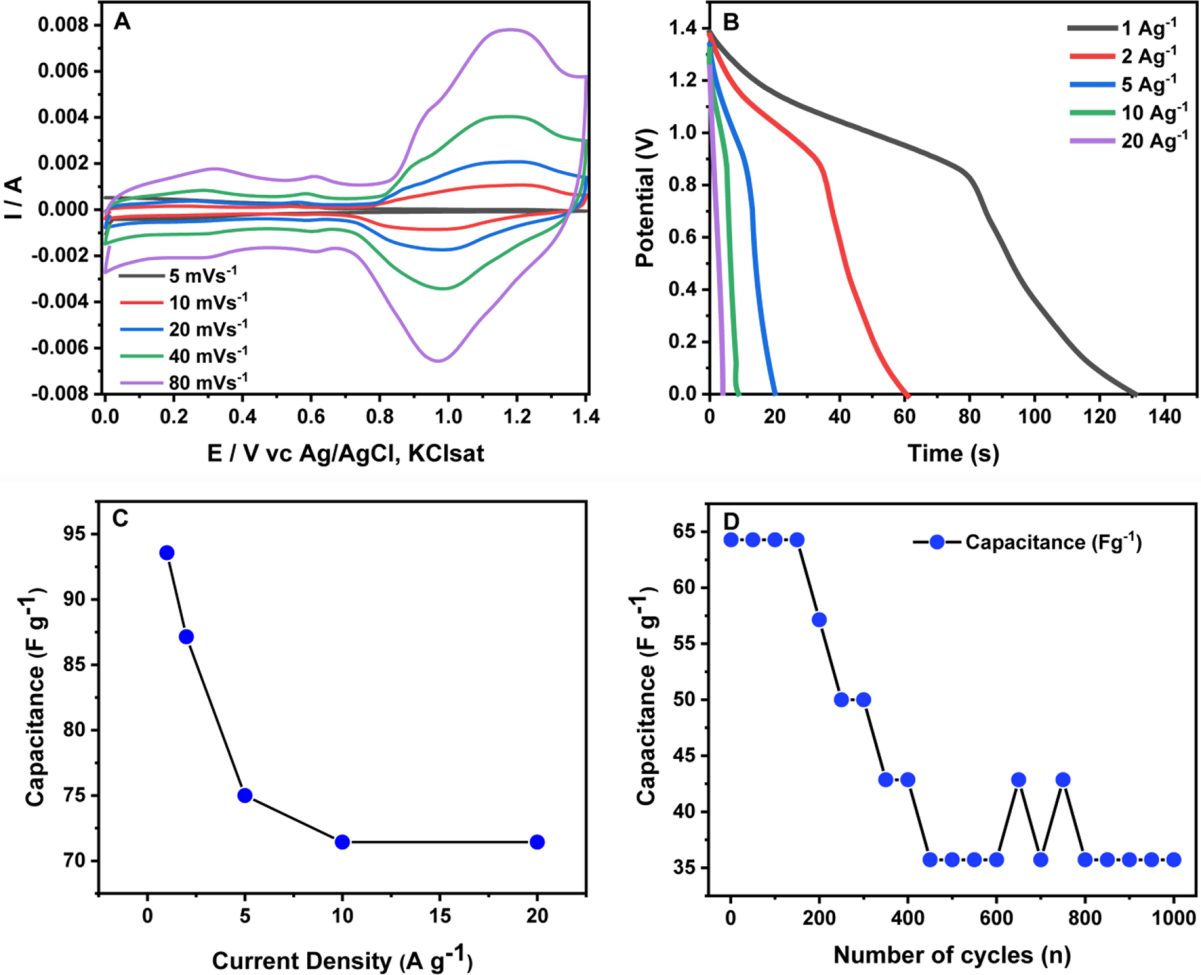
**A** The hybrid system's CV curves at different scan rates (2.0 molL^−1^ KOH electrolyte) and **B** charge–discharge profiles at 1, 2, 5, 10, and 20 A g^−1^. **C** Specific capacitances vs. current densities. **D** Stability after 1000 cycles at 10 A g.^−1^

As a final evaluation of the Zn-doped MnO_x_ nanowires' performance, we assembled a hybrid supercapacitor, using our material as the positive and activated carbon as the negative electrodes. The possible operating voltage window of the hybrid device was studied by CV (Fig. 8A). The optimum voltage window was 0.0 to 1.4 V vs. Ag/AgCl, KCl_sat_ electrode. Replacing the values obtained from GCD analysis in Eq. 4, we reach a mass balance ratio of 5.9, in other words, 1:5.9 in the 2.1 wt.% Zn-doped nanowires and activated carbon, respectively. Performing CV curves at different scan rates showed a broadening of redox peaks due to a combination of supercapattery and capacitive electrodes. Also, one can notice the high-rate capability of the hybrid supercapacitor once scan rates from 5 to 80 mV s^−1^ kept the CV curved unchanged. The charge–discharge curves for the hybrid device were obtained under the same conditions for the Zn-nanowires nanowires (Fig. 8B-C), and the data obtained were: 93.6 F g^−1^ (1 A g^−1^), 87.1 F g^−1^ (2 A g^−1^), 75.0 F g^−1^ (5 A g^−1^), 71.4 F g^−1^ (10 A g^−1^), and 71.4 F g^−1^ (20 A g^−1^). The Coulombic efficiencies were higher than 90%. The hybrid device yielded a maximum energy density of 91.7 Wh kg^−1^ at a power density of 2520 W kg^−1^ at 1 A g^−1^. The energy density obtained here is higher than conventional electrochemical double-layer capacitors, which is highly interesting for future applications [41]. We also performed 1000 cycles for the hybrid system, which showed a capacitance decrease of up to 250 cycles, with stabilization to 35 A g^−1^ up to the end of the 1000 cycles (Fig. 8D).

The comparison of energy density and power density of assembled ASCs with other devices from the literature is shown in Table 1. Previous research has suggested that MnO_2_ or ZnO-based electrodes can enhance energy storage performance. Our findings were comparable but, in some cases, surpassed those reported recently. It's important to acknowledge that we know the limitations that need to be addressed, particularly concerning stability. Nonetheless, it is noteworthy that our study, for the first time, unveiled the connection between oxygen vacancies and the cross-section of nanowires with the storage capacity of MnZn nanowires-based materials.

**Table 1:** Comparison among the present work and some from the literature

Material	Structure	Energy density (Wh kg^−1^)	Power density (W kg^−1^)	Ref
Zn-doped MnO_x_	Nanowires	91.7	2520	This study
MnO_2_/ZnO	Nanowires/nanorods	55.28	950	[42]
ZnO	Nanorods	36.45	28.16	[43]
ZnO/MnO_2_	Nanorods/nanowires	12	4790	[44]
α-MnO_2_@δ-MnO_2_	Nanowires/nanosheets	78	21.7	[45]
MnO_2_@NiCo_2_O_4_	Nanowires	35.6	745.1	[46]
N-doped MnO_2_	Nanowires	23.7	2000	[47]
NiO/ZnO	Nanowires	23	614	[48]

## Conclusions

Herein, we prepare one-dimensional Zn-doped nanowires with a simple hydrothermal synthesis method, varying the quantity of Zn content. The material presented a high specific capacitance of 1082.2 F g^−1^ at a charge/discharge current density of 1.0 A g^−1^ in 2.0 mol L^−1^ KOH electrolyte with an optimized Zn content, which is a remarkable result compared to the literature. Surprisingly, we found a volcano-type relationship between the performance and the Zn loading, demonstrating that the highest performance storage material could be achieved by incorporating 2.1 wt.% Zn-doped MnO_2_ nanowires. Then, adding Zn ions was essential, even in a very low concentration. Interestingly, zinc sites could be uniformly dispersed onto α-MnO_2_ nanowires structure as a function of composition (0.3, 2.1, 4.3, and 7.6 wt.% of Zn), avoiding the formation of different crystalline phases or depositing zinc particles at the surface of the α-oxide nanowires. The EPR results showed that oxygen vacancies achieved a higher value for the optimized material, which could help to explain its performance. More importantly, a hybrid capacitor based on the Zn-dope nanowires and activated carbon electrodes supplied an energy density of 91.7 Wh kg^−1^ and a power density of 2520 W kg^−1^ at 1 A g^−1^. Once the synthesis of the Zn-doped nanowires is simple and one-pot, we believe this material can be a promising electrode for low-cost storage devices.

### Supplementary Information

**Additional file1** Additional data regarding the material characterizations and performance can be found in the supplementary file

## Data Availability

The data that support the findings of this study are available from the corresponding author, M.A.S.G, upon reasonable request.
