# Supplementary material for: Zn‐doped MnOx nanowires displaying plentiful crystalline defects and tunable small cross-sections for an optimized volcano-type performance towards supercapacitors
Source: Discov Nano. 2023 Dec 4;18(1):147. doi: 10.1186/s11671-023-03933-2 (PMC10695906; doi:10.1186/s11671-023-03933-2)
Supplement: Supplementary file 1 — Additional file1 Additional data regarding the material characterizations and performance can be found in the supplementary file [file 11671_2023_3933_MOESM1_ESM.docx]

**Zn‐Doped MnO_x_ Nanowires Displaying Plentiful Crystalline Defects and Tunable Small Cross-sections for an Optimized Volcano-type Performance Towards Supercapacitors**

Geyse A. C. Ribeiro,^1^ Scarllett L. S. de Lima,^2^ Karolinne E. R. Santos,^1^ Jhonatam P. Mendonça,^1^ Pedro Macena,^2^ Emanuel C. Pessanha,^2^ Thallis C. Cordeiro,^3^ Jules Gardener,^4^ Guilhermo Solórzano,^2^ Jéssica E. S. Fonsaca,^5^ Sergio H. Domingues,^5^ Clenilton C. dos Santos,^6^ André H. B. Dourado,^7^ Auro A. Tanaka,^1^ Anderson G.M. da Silva,^2,*^ Marco A. S. Garcia^1,*^

*^1^Departamento de Química, Centro de Ciências Exatas e Tecnologia, Universidade Federal do Maranhão (UFMA), São Luís – MA, Brazil.*

*^2^Departamento de Engenharia Química e de Materiais-DEQM, Pontifícia Universidade Católica do Rio de Janeiro (PUC-Rio), Rio de Janeiro – RJ, Brazil.*

*^3^Centro de Ciências Exatas e Tecnologia, Universidade Estadual do Norte Fluminense Darcy Ribeiro (UENF), Rio de Janeiro – RJ, Brazil.*

*^4^Center for Nanoscale Systems, School of Engineering and Applied Sciences, Harvard University, Cambridge, USA.*

*^5^Mackenzie Institute for Advanced Research in Graphene and Nanotechnologies – MackGraphe, Mackenzie Presbyterian University, São Paulo – SP, Brazil.*

*^6^Departament of Physics, Universidade Federal do Maranhão (UFMA), São Luís – MA, Brazil.*

*^6^São Carlos Institute of Chemistry, Universidade de São Paulo (USP), São Carlos – SP, Brazil.*

*Corresponding author. Email: [agms@puc-rio.br](mailto:agms@puc-rio.br), [marco.suller@ufma.br](mailto:marco.suller@ufma.br)


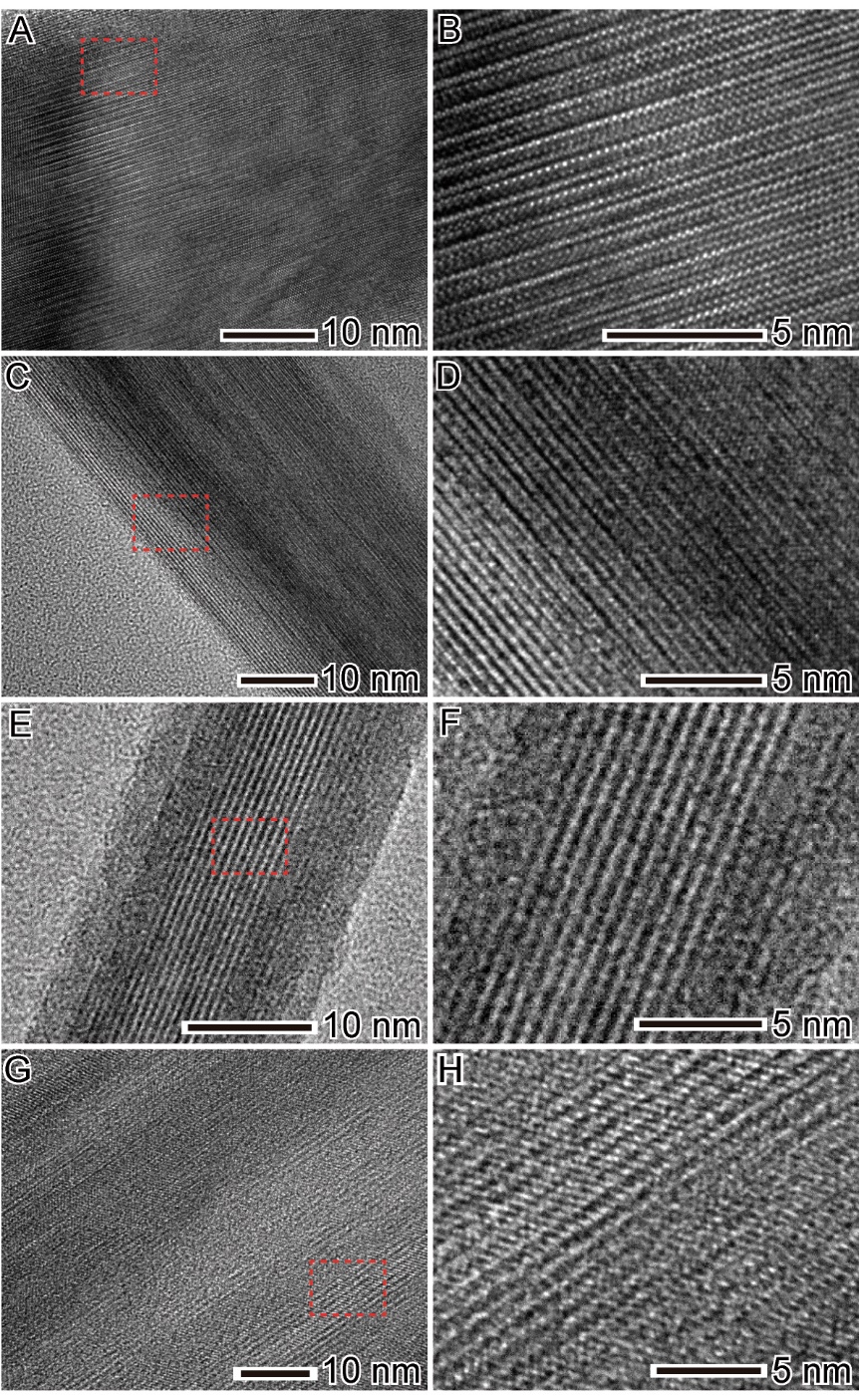


**Figure S1.** HRTEM images of 0.3 wt.% (A-B), 2.1 wt.% (C-D), 4.3 wt.% (E-F), and 7.6 wt.% (G-H) Zn-doped MnO**_x_** nanowires.

| Catalyst | Specific Surface Area (m^2^/g) | Total Pore  Volume (cc/g) | Average Pore Diameter (nm) |
| --- | --- | --- | --- |
| MnO_2_ | 105 | 0.05 | 2.5 |
| 0.3 wt.% Zn-doped MnO_x_ | 107 | 0.06 | 2.4 |
| 2.1 wt.% Zn-doped MnO_x_ | 132 | 0.08 | 2.3 |
| 4.3 wt.% Zn-doped MnO_x_ | 144 | 0.07 | 2.4 |
| 7.6 wt.% Zn-doped MnO_x_ | 137 | 0.06 | 2.4 |

**Table S1.** Textural properties measured by N_2_-physisorption for the MnO_2_ and Zn doped-MnO_2_ nanowires.


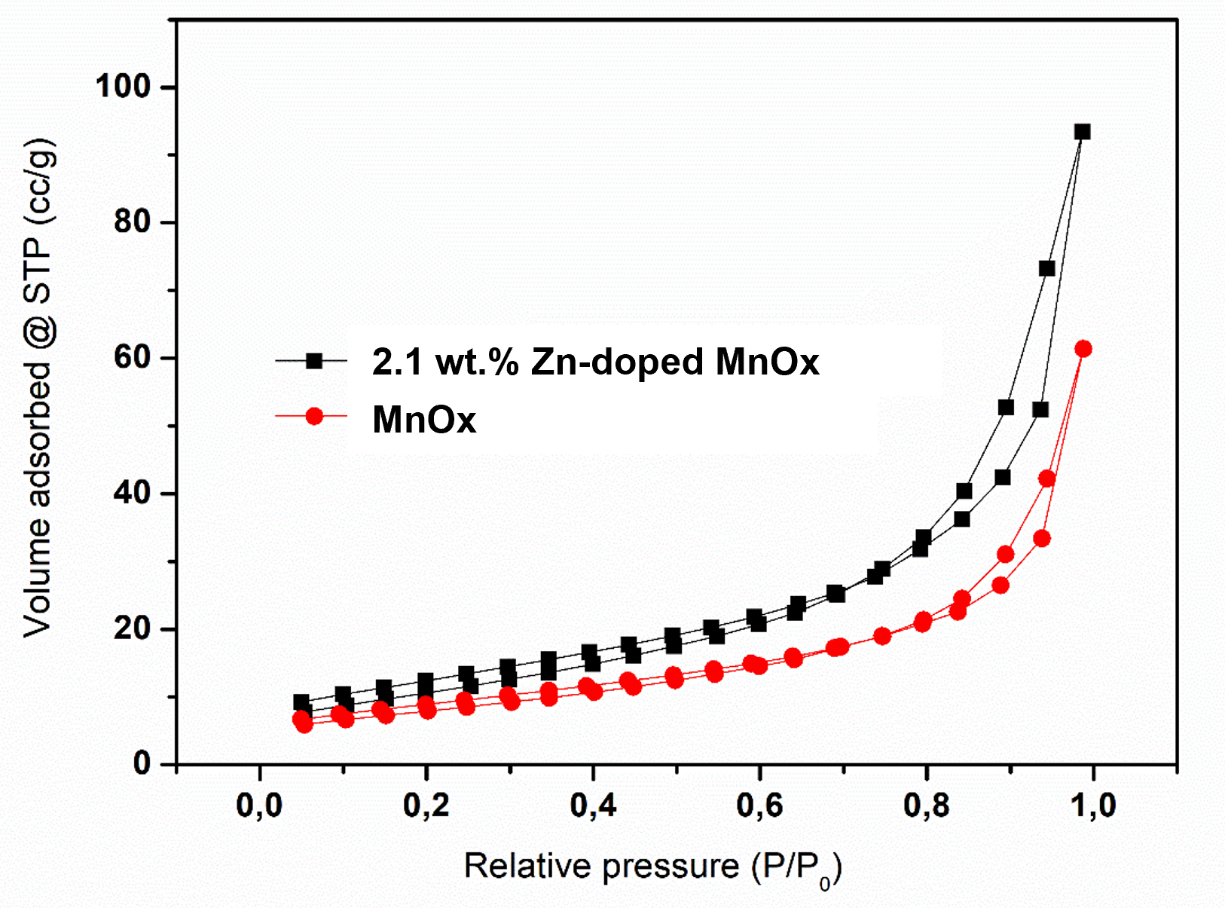


**Figure S2.** Typical N_2_-Adsorption/desorption isotherms for MnO_x_ and Zn-doped MnO_x_ nanowires. All the doped materials showed similar isotherms; thus, for clarity, just the 2.1 wt**.**% Zn-doped MnO**_x_** sample is shown.


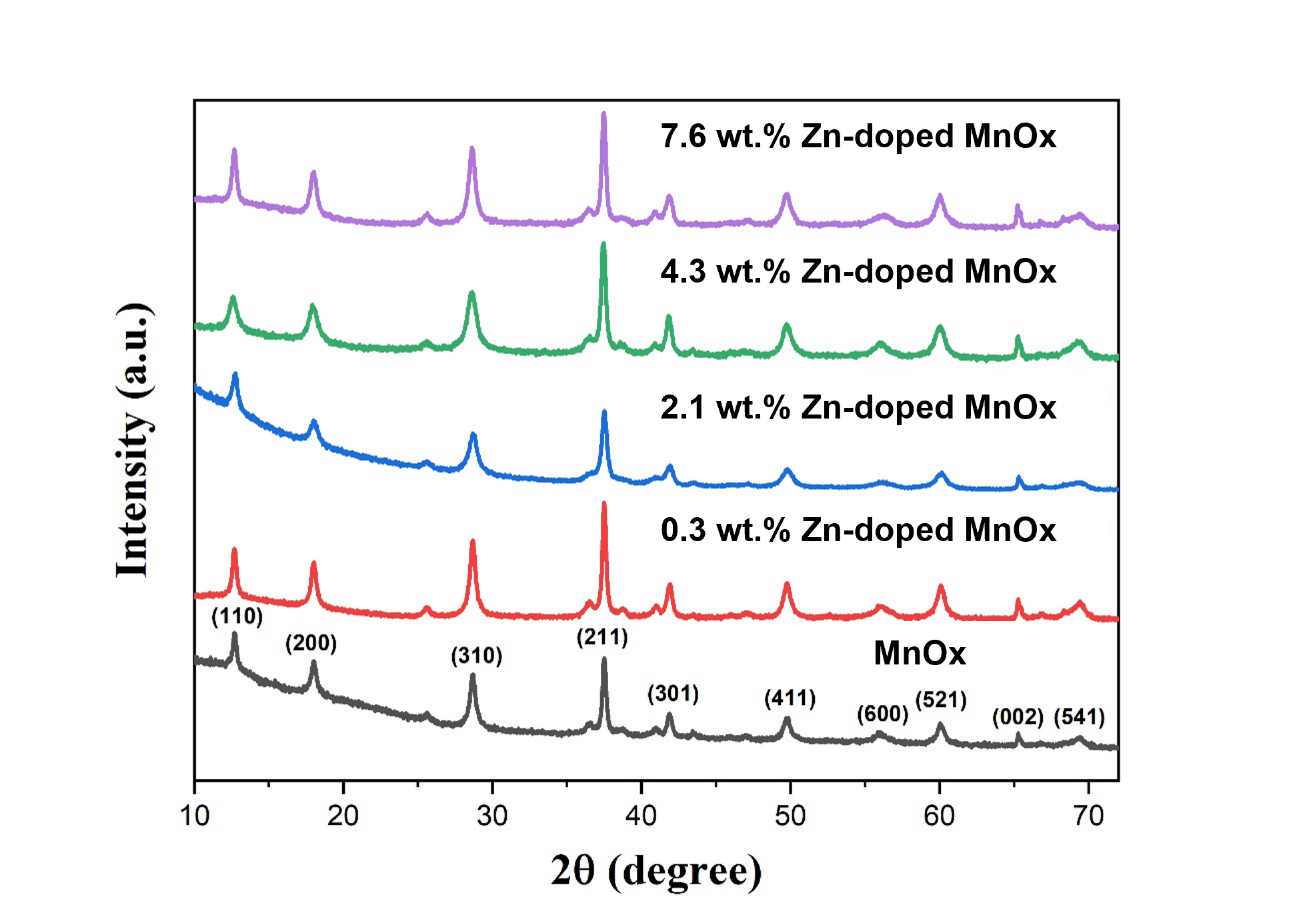


**Figure S3.** XRD patterns of MnO_x_ and Zn-doped MnO_x_ nanowires.


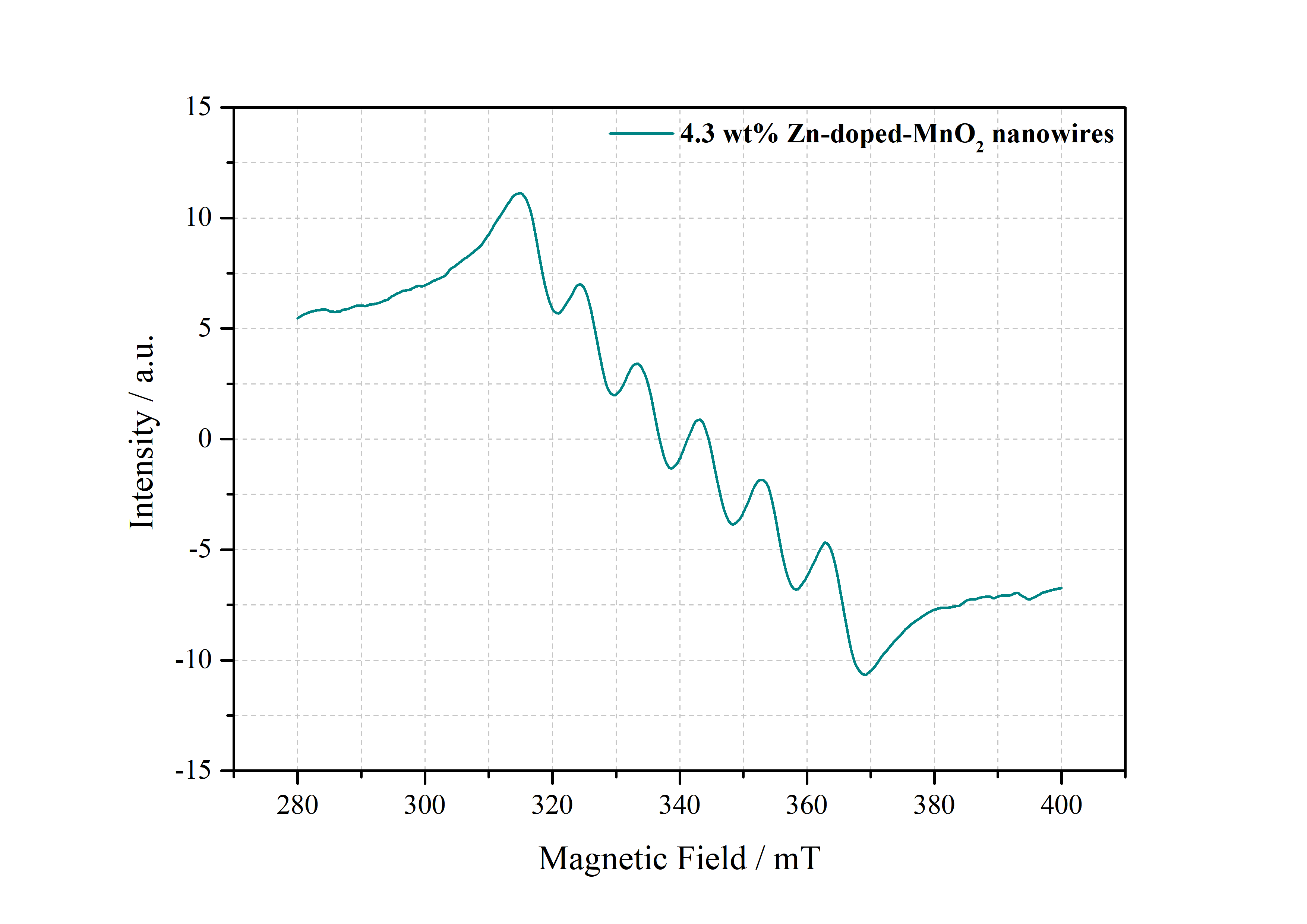


**Figure S4.** Sextet EPR signal for the 4.3 wt.% Zn-doped MnO_x_ nanowires.

**
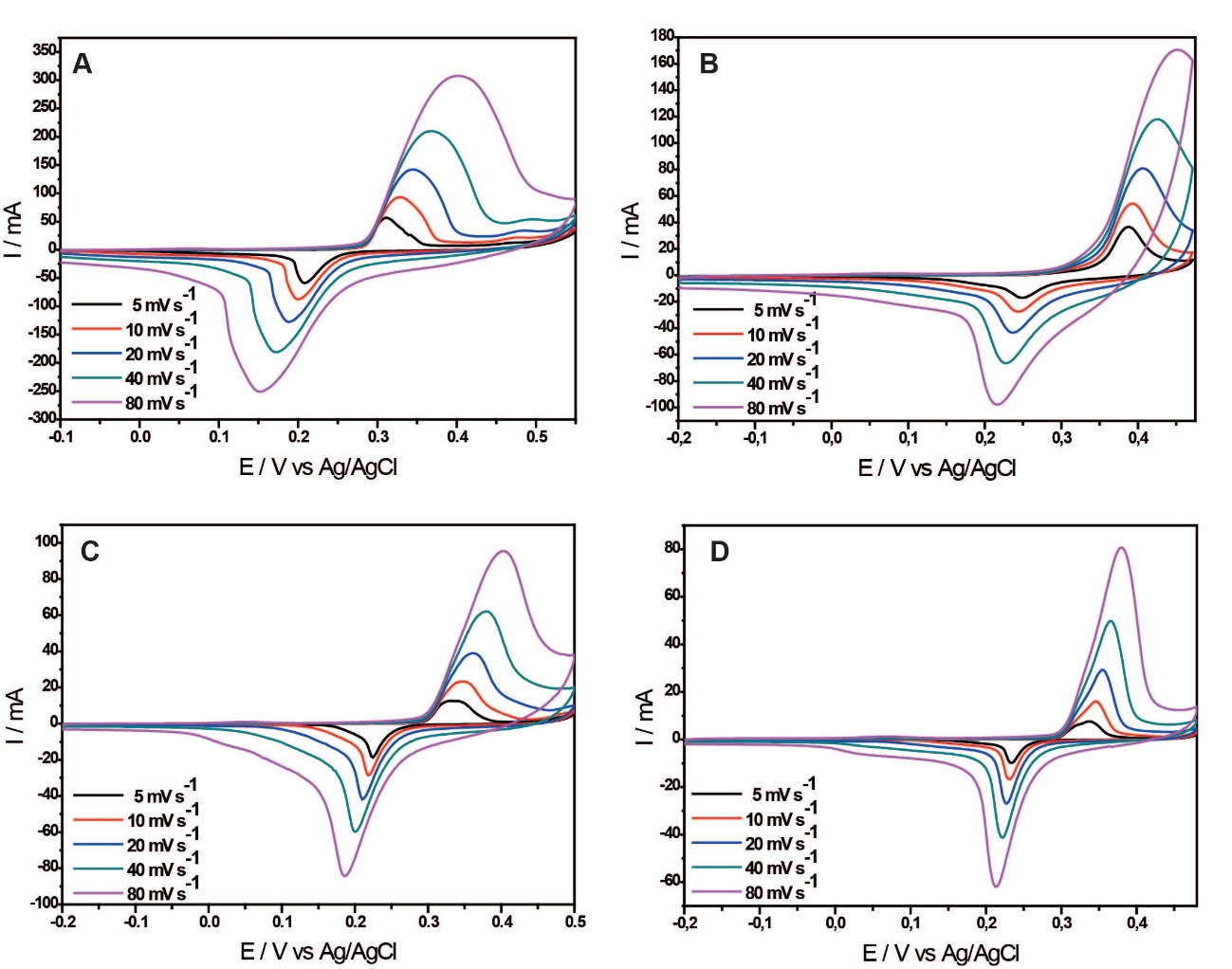
**

**Figure S5.** CV curves of the A) 0.3 wt%, B) 2.1 wt.%, C) 4..3 wt.%, and D) 7.6 wt.% Zn-doped MnO_x_ nanowires at different scan rates.

**
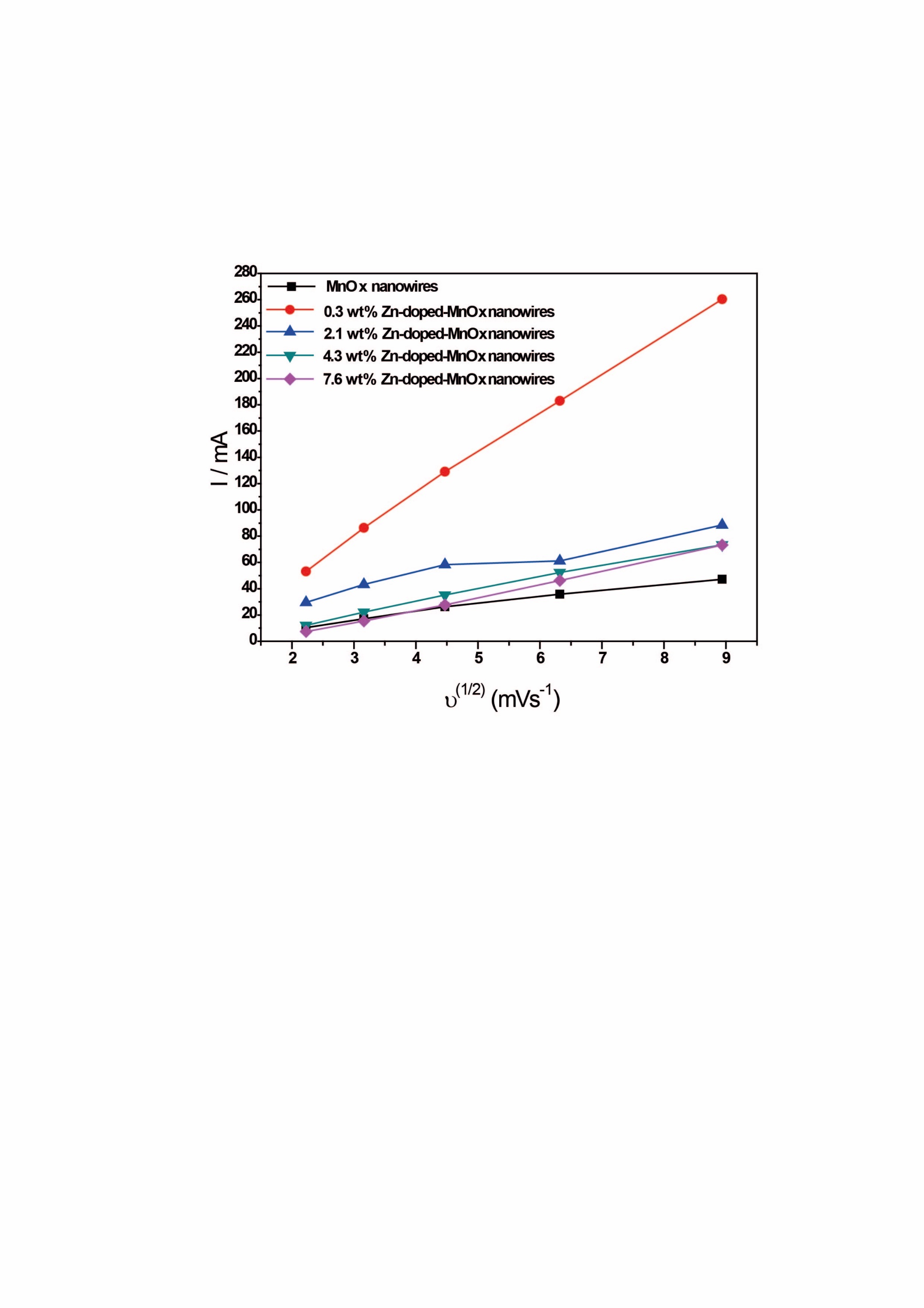
**

**Figure S6.** Current density vs. square root of the scan rate for the MnO_x_ nanowires-based electrodes (doped and undoped) in the KOH 2.0 M.

**
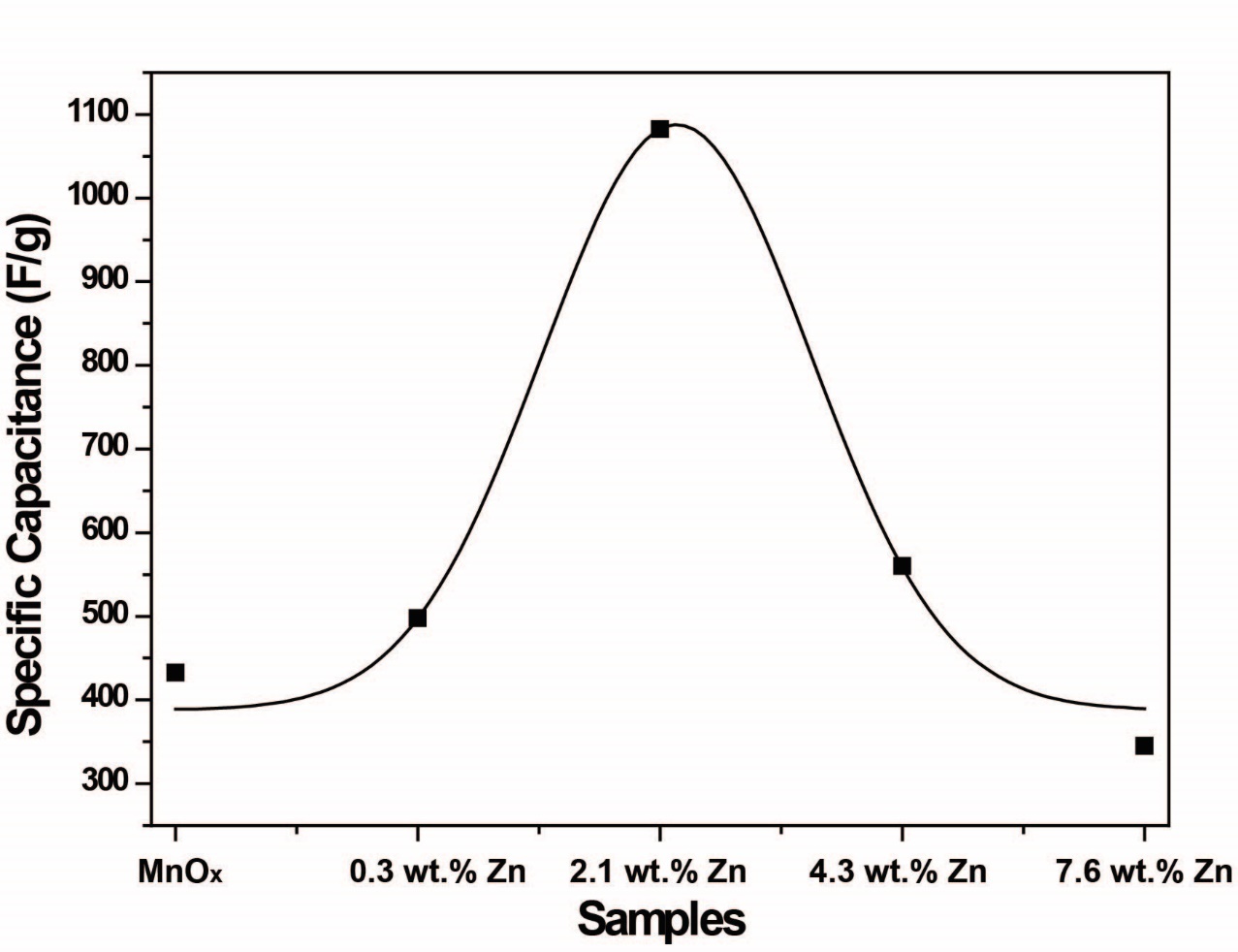
**

**Figure S7.** Volcano-like relationship between the storage performance and the Zn loading.


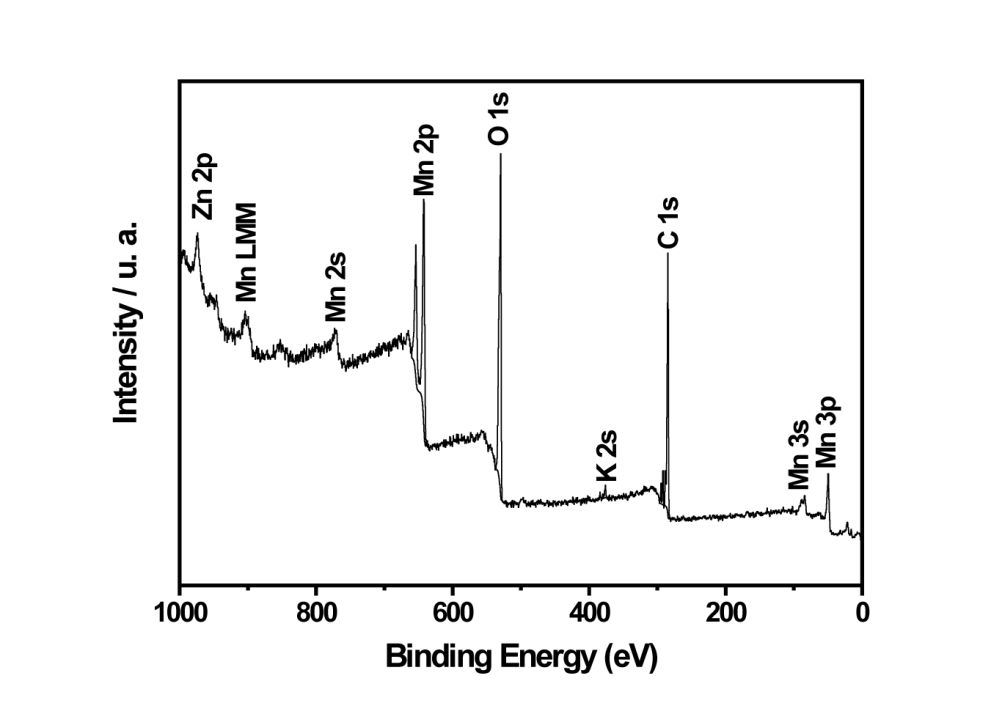


**Figure S8.** Survey spectra of the 2.1 wt.% Zn-doped MnO_x_ nanowires.

**
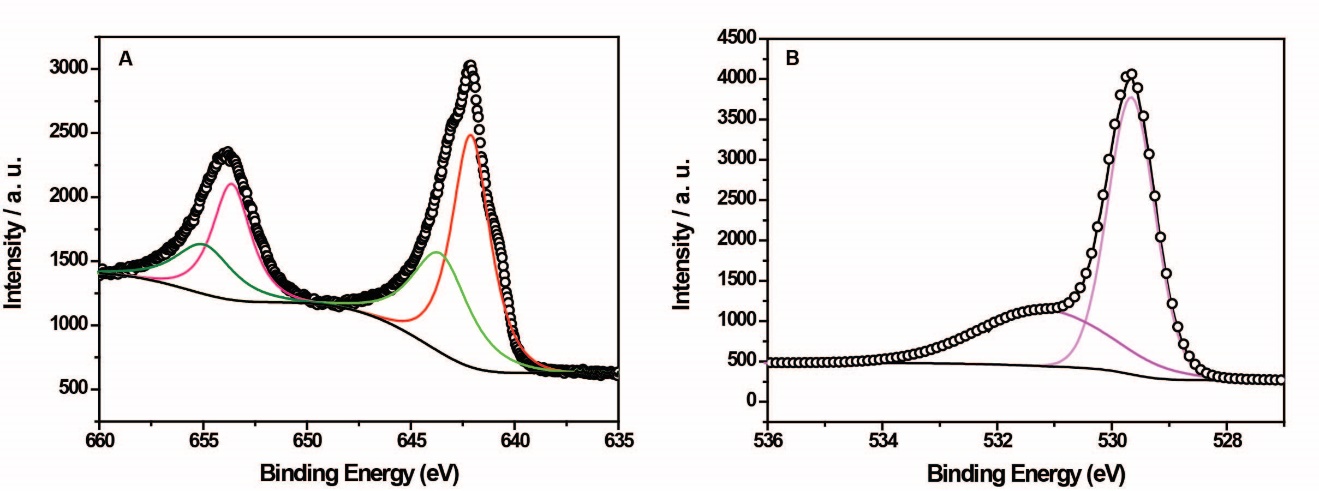
**

**Figure S9.** High-resolution XPS spectra of A) Mn and B) O for the MnO_x_ nanowires.
